# Connecting worlds: social work educators’ perceptions on the role of lived experience in pedagogic practice

**DOI:** 10.1080/02615479.2022.2142548

**Published:** 2022-11-11

**Authors:** Suzanne Sayuri Ii, Hannah Jobling, Kelly Devenney, Sue Ziebland

**Affiliations:** a Nuffield Department of Primary Care Health Sciences, University of Oxford, Oxford, UK; b Department of Social Policy and Social Work, University of York, York, UK

**Keywords:** Social work education, lived experience, qualitative methods, pedagogy

## Abstract

This qualitative study explores social work educators’ perceptions on the role of lived experience in teaching within undergraduate and postgraduate social work education programmes across universities in Britain. Thirty-five semi-structured online interviews were conducted with social work educators from 27 universities across Scotland, Wales and England. Findings were that educators indicated specific ways that people with lived experience (PwLE) can transform student learning. They give an opportunity to expose students to different perspectives, challenge stereotypes about people who access services, help students reflect on their own personal and professional values, demonstrate that the curriculum is connected to and grounded in the real world, and provide crucial preparation before practice. While the extant literature highlights the positive benefits of PwLE involvement in higher education programmes, ongoing work is required to support PwLE involvement consistently and sustainably, and to ensure more diverse representation of PwLE in order that students are exposed to a broader, real world understanding of practice.

## Background

Social work educators increasingly involve people with lived experience of services (PwLE) in the delivery of social work education (McLaughlin et al., 2016). This approach emerged primarily within the UK context, where it has been underpinned by a statutory requirement to involve PwLE in the design and delivery of qualifying programmes since 2003 (Department of Health, 2002). This practice is becoming more widespread internationally, supported by a growing literature (Cabiati et al., 2021; Ramon et al., 2019) which suggests that involving PwLE can benefit student learning, improve social work practice and have positive effects for those PwLE who choose to participate (Irvine et al., 2015; Robinson & Webber, 2013).

### How have PwLE been involved in social work education?

The research literature references a broad range of activity by PwLE, mostly related to direct involvement in teaching, but also covering admissions (Rooney et al., 2016); assessing students’ practice and written work (Pearl et al., 2018); placement provision and project work (Gee et al., 2009); and the design and oversight of social work programmes (Webber & Robinson, 2012). Involvement in teaching specifically may include contributing testimony to lectures (Sapouna, 2021); leading workshops (Duffy, 2012); engaging with students in dialogic activities and collaborative projects (Levy, 2016); partnering in role plays (Duffy et al., 2021); and contributing to digital and audio-visual materials such as videos, podcasts, blogs and documentaries (Lucas & Thomas, 2021).

Despite the UK’s long-established tradition of involving PwLE however, there remain significant variations between institutions in the extent and depth of involvement (Hatton, 2017). Previous studies report difficulties in reaching meaningful involvement rather than tokenism (Molyneux & Irvine, 2004), including organizational boundaries to meaningful involvement of PwLE (Gutteridge & Dobbins, 2010) and lack of wider institutional support to involve PwLE in social work partnership models (Taylor & Le Riche, 2006). While there are reported challenges in the literature around PwLE involvement, there are benefits to students in social work higher education programmes.

### What have evaluations of PwLE involvement in social work education shown?

Research on student perceptions and experiences suggests two interrelated benefits; an expansion and consolidation of their social work knowledge and skills, and opportunities to challenge and explore their social work values. Unwin et al. (2017) reported that involving PwLE transformed student perceptions to dispel myths of disability, encourage empathy, and challenge stigmas that may act as barriers to relationship building. Direct interactions between students and PwLE also give students opportunities to demonstrate and consolidate specific skills, such as communication skills (Anghel & Ramon, 2009; Irvine et al., 2015; Skilton, 2011) and there is some evidence that the visceral nature of exposure to people’s ‘lived experiences’ can make learning more memorable (Agnew & Duffy, 2010; Lane et al., 2010). The majority of studies usefully evaluate the experiences of students and PwLE in relation to individual pedagogical projects, many of which have been synthesized in literature review or research overviews (Chambers & Hickey, 2012; Robinson & Webber, 2013; Wallcraft et al., 2012). More recently, there has been a concern to assess the impact of including PwLE in social work education on student learning and ensuing practice (Hughes, 2017).

Although much of the extant research concerns pedagogical projects designed and delivered by social work educators, little has been published about educators’ own perceptions of the involvement of PwLE, with focus being on the perceptions and experiences of students and PwLE themselves. In the UK, PwLE have helped guide the design, delivery and review of social work curricula for two decades. This offers an opportunity to invite educators to reflect on this practice. In this paper we contribute an analysis of the perspectives of UK based social work educators, which explores how they see their role in bringing PwLE and students together and what they hope to achieve when they involve PwLE into their teaching.

## Methods

### Study design and setting

We report the findings of a qualitative, semi structured interview study with social work educators in Britain. We invited social work educators to take part in an interview to discuss how accounts from PwLE are integrated into their teaching, and their views about what (if anything) they think that students learn from this inclusion.

### Participant recruitment

We invited educators who currently teach on a higher education programme in social work. Some participants also had responsibility for coordinating contributions from people with lived experiences, but all were experienced teachers. A recruitment flyer was distributed through Social Work England with an encouragement to share with interested parties. Potential participants contacted the lead researcher SSI to express interest.

With informed consent, thirty-five participants were interviewed February-April 2021; four worked in Scotland and one in Wales, while the other 30 taught in England. Educators from 27 universities participated, 26 were female and 9 were male. Participants had a mixture of teaching experience on undergraduate, postgraduate, and/or post-qualifying programmes in social work. There were a small number of participants who also taught in health and social care; a small number of participants coordinated teaching activities with PwLE groups for the programmes, or recruited PwLE individuals or organizations to the programmes, in addition to their teaching responsibilities. The numbers of PwLE who were attached to programmes throughout the country were variable; PwLE member numbers were in single digits ranging from 5–8 members for some programmes, while other programmes had nearly 100 PwLE members. Participants were sent a digital copy of the signed consent form and one digital copy was recorded and stored on a University server. The research project was approved by The Central University Research Ethics Committee (CUREC) at the University of Oxford (Ref Number: R64616/RE001).

### The interviews

SSI, a social scientist experienced in qualitative research, conducted the interviews online (due to the Covid 19 pandemic). Interviews were semi-structured with prompts and probes, lasting from 21 minutes to 58 minutes. Field notes were written during and after the interview to document the context. The interview guide began with questions about how people’s lived experiences had been integrated into the participants’ teaching. We also asked about the range of approaches they had used and invited them to reflect on what they thought might be achieved through including lived experiences in teaching on social work modules. The list of semi-structured ‘prompts’ for the interviews was discussed with coauthors including the PI, a senior qualitative researcher SZ.

### Data analysis

Interviews were audio recorded, transcribed verbatim and identifiable information redacted from the transcriptions. Interview audio files were deleted immediately after transcripts were received and reviewed. Raw transcripts were password protected and only accessed by the lead researcher (SSI). Transcripts with redacted identifiers were used for data analysis and were only accessible by the research team. Transcripts were uploaded to QRS NVivo, version 12 (QSR International Pty Ltd., Doncaster, VIC, Australia). The ‘One sheet of paper’ (OSOP) approach (Ziebland & McPherson, 2006) was used to produce mind maps of the theme ‘PwLE involvement and teaching’ and the different issues raised through the coded extracts from the respective section of data. This process was based on constant comparison with each interview transcript, to interrogate the themes discussed in this paper, which were developed with the coauthors.

*Note*: This literature uses a range of descriptors for people who contribute to social work education, most often in the UK context, ‘service users and carers’. In this article we use the phrase ‘people with lived experience’ (PwLE) in recognition of the well documented concerns about the term ‘service user’ in particular (McLaughlin, 2009), and to emphasize the centrality of experiential knowledge in social work education (Duffy & Beresford, 2020). In the results, there are multiple terms used by participants when they refer to PwLE.

## Results

In the interviews, educators presented a plethora of reasons for integrating people’s lived experiences of social work teaching with the aim of supporting students’ learning and professional development. These include the value of being exposed to different perspectives, providing evidence that the curriculum is connected to and grounded in the real world, preparing the students for placements and (sometimes) challenging preconceptions about people who access care. These themes are discussed below and illustrated with excerpts from the interviews.

### Exposure to different perspectives

The educators described social work as a discipline that draws on multiple sources of knowledge and other academic disciplines (e.g. psychology, sociology, law). Social work courses integrate perspectives from social work practitioners, lawyers, health professionals, as well as people with lived experience. In this sense, social work lecturers mediate various forms of knowledge to expose students to different experiences and perspectives on the forms and functions of social work. While incorporating the approaches and theoretical perspectives of other disciplines, some educators emphasized that the central and critical point was for the students to understand the experiences of the person (and family) they would work with.

So, I suppose what we’re trying to do is … that it’s not just us as lecturers who hold the knowledge in social work. You know we can guide people toward it who are here at the university, but really, it’s, it’s about that shared ownership, the shared story of what social work is as told by people who themselves have experienced it. (Participant 34, Assistant Professor)

If I were to find a silver lining between most of the sessions or all the things that we do in the course, [it] is always getting students to understand the perspective of the service user and what their expectations are from a professional, and what would work better for them in different situations. If it’s Child Protection or Adult Care or something else. So, it’s always bringing in the other side, trying to see whatever you’re learning about, for example, Mental Capacity Act from the perspective of the person that actually has to live with your decision as a social worker. (Participant 11, Senior Lecturer)

Educators stressed the importance of introducing students to a broadly ecological understanding of the individual (and their family), including influential environmental and societal mechanisms, and the social and psychological drivers that are embedded in an individual’s life circumstances. It could be challenging for students to acknowledge the validity of multiple perspectives and to grasp the complexities of working with people throughout the life course. The exposure to interdisciplinary, inter-sectoral and inter-professional perspectives should (it is hoped) help the student to develop their skills as a compassionate and effective practitioner.

Therefore, you need to have multifaceted forms of knowledge and understanding, which can lead to—fundamentally, social work is about concern for human wellbeing and human suffering in all of those forms … And in order to understand that, social workers operate both with individuals and families, but also understanding that person’s context. And in order to do that, you have to have an understanding about how society functions. And obviously the laws and policies that create the environment in which people that we work with live, and ourselves. So, but it’s also about having … engendering a sense of compassion that people’s life circumstances are often dictated by things beyond their own control. But it’s about empowering people to take control of what they can and understand the situation that people are in. (Participant 2, Programme Lead, BA and MA)

Educators suggested that there was particular value in hearing lived experience which could be more powerful (and challenging) for the students than information conveyed in a conventional lecture.

[There’s no] one way of understanding social work and it doesn’t come always from the professional perspective— if they’re [students] really gonna understand what it is to be a social worker, they need to understand who has got something to say about social work as a practice. What makes a good social worker … But [for example], I’ll [PwLE] tell you what it feels like … and that punches people in the stomach … We can teach and we can stand up and lecture, but it doesn’t reach them in that emotional, kind of, visceral way … Someone with their own kind of lived experience can reach out. (Participant 12, Programme Lead, MSc)

### Grounding the curriculum in the real world

Theoretical concepts can be difficult for students; some educators explained that by drawing on and connecting theory to real life experiences they were better able to demonstrate and ground the relevance of the key concepts, for example of moral philosophies which can be ‘hard to digest’ without examples:

So … we translate it all … theory into concrete life examples from her life [of a PwLE] and [the] life of her boy. And students get it better … They’re … more ready to understand … [the] moral imperative of respecting human beings […] So, that’s one way translating concepts into real life and … developing their capacity to think … theoretically about something. (Participant 11, Senior Lecturer)

A few educators described dialogs with students about different models of practice and approaches to support individuals, as examples of the diversity of perspectives. Without an understanding of ‘the lived realities of people … then we are really just completely stamping our authority or a pre-conceived idea onto somebody else that could be quite harmful’ (Participant 8, Senior Lecturer).

Educators cautioned against applying theories, models or perspectives before considering the individual and their circumstances:

I think it’s really important when we’re teaching about theory and is... to remind students that you’ve gotta see the person first and you’re not going in with the theory to think, right, how am I going to apply this theory to this individual or to this situation. It’s, how am I going to try and understand this individual? And perhaps looking through the lens of different theories might help me to understand. But I’ve [as the social worker] got to be led by them [the individual] … because otherwise it becomes contrived, doesn’t it, and you’re trying to squeeze a situation. You’re trying to make it fit what you think the theoretical knowledge is telling you. (Participant 30, Programme Lead, MA)

Educators discussed the negative image of the social work field in the UK and historically oppressive practices and the need to address professional practice in a balanced light. Educators were able to draw on a range of resources and materials, as well as directly from PwLE, to illustrate examples of good and poor practice in social work and the implications for the individual.

So, the reality of [social work] practice [is] that ... this is very important work to do. That they [practitioners] have power and the power that goes with the social work qualification or for those who have a social care degree, are then licensed ... to work with very vulnerable people, individuals, groups. [The practitioners] are in powerful positions and that power needs to be acknowledged and used positively because … it can be used in other ways. And I think that’s really important for, for students to appreciate because power is often associated negatively ... in sort of social narrative. And I think this helps to establish that … positive messages, messages about social work as a profession and its responsibility in the roles and values…Listening to narratives … the lived experiences of people to bring this to life that reinforces the significance, the power, the need to do the right thing. … I think this all reinforces the value base of the profession that we try to instill in the students. (Participant 8, Senior Lecturer)

### Preparation for placements

Students need to be prepared for their social work placements, which occur multiple times during their course and are designed for them to gain (supervised) experience in the field. Before their first placement, students may have very limited experience of interacting with a PwLE of social work services. Interacting with PwLE from the beginning of the programme helps students ‘to feel a bit less anxious about going out and working with folk when they build those relationships with folk before they go out on placement’ (Participant 12, Programme Lead, MSc). Students ‘get a bit nervous and feel like they are at risk of bothering service users so it breaks down that barrier a little bit and gives them practice’ (Participant 23, Graduate Teaching Assistant) before their placements.

Social work educators are often former (rather than current) practitioners and likely to be aware that they are no longer able to rely on recent, first-hand experiences from the field. PwLE can, through their direct accounts, help to reinforce the immediacy and credibility of what is taught.

We’re all social, all ex-social workers [on the teaching team]. None of us have practiced for quite a while because the longer you stay as an academic, the less likely you are to be in practice. So, it’s almost as soon as you walk through that university door, you start to lose your credibility as a practitioner because you’re becoming something else. And so, as I can’t provide 100% credibility as a practitioner, the input of service users is really important for me to enhance that. (Participant 13, Senior Lecturer)

To help the students prepare, some educators told us that they worked with a PwLE as a guest lecturer, drawing on their help for assessment activities, or through role plays or simulations. Educators stressed the importance of creating a supportive environment for the students to actively listen, discuss and learn from their interactions with a PwLE as a valuable introduction to the real world of social work. For some students, the involvement of PwLE in the classroom is their first experience interacting with people who access social work services. Educators described the supportive environment of the classroom as a safe space that gives the students an opportunity to learn through self-reflecting on their actions, communication skills and the language they used. The educators and PwLE can encourage discussion and help them *learn from mistakes* to build on introspection and awareness in a supportive environment.

The safety (supportive environment of the classroom) aspect is crucial because I want students to be able to take the opportunity to not just hear these narratives and experiences, but also engage with them, to be switched on by them. Have the opportunity to ask questions and the safe environment is important because … I hope that they can make mistakes within that environment as well and say the wrong thing because they’re gonna do that in practice and it’s through making those mistakes and maybe using the, the wrong language sometimes, as an … example. (Participant 9, Head of Department)

Social work educators explained that building relationships, partnerships and alliances with people who access social work services is key to social work practice. Good communication skills are needed to approach individuals and interact with them respectfully. When students hear PwLE talking openly and in detail about their (perhaps traumatic) experiences, the educator can help to foster an environment where difficult and uncomfortable subjects can be broached without causing enervating fears of getting it wrong or causing offense. When a PwLE is physically present in the classroom, rather than represented through a recording, they may also help to guide these difficult discussions and provide direct feedback on communication.

Educators and PwLE also coordinated activities for PwLE to act out role plays and simulations to give students practical experience in the classroom. Through role plays, students communicate and interact with a PwLE who is acting out a role that is based on a case study. In simulations, students may enact their role as a social worker in a mock-up of a community environment with a PwLE playing the role of the client. Both activities are supervised and, in some cases, recorded to review and revise the student’s behavior, communication and approach.

In order to then go out on placement though, they [the students] have to, they have to be able to show that they can engage with people. So, actually, they’ve already got to have shown a certain ability and certainly skills before they go out there. So, it’s about actually having an environment within the university where that can be assessed and observed. So, it is very much part of seeing their professional skills in action. Effectively, before you kind of let them loose out in, even though they’re supervised in placement, it’s still before they then go into a genuinely real-life situation, even though it’ll be with support and with practice educators and link workers on placement, you need to have a more controlled environment in which you’re then seeing what they do … So, it’s to try and kind of connect those two worlds together. (Participant 15, Senior Lecturer)

### Challenging students’ ideas about people who access social work services

Students sometimes need to learn to suspend or question their own assumptions, biases or stereotypes about people who access social work services. Teaching an ethical code of practice can sometimes include deconstructing student’s ideas about people who access social work services. Hearing from people with lived experience can help to address misconceptions, myths and/or stereotypes, and help to understand and challenge the position of power that comes with a professional degree or qualification. When students are able to access a range of resources with different accounts of lived experiences, they may avoid the neophyte error of making unwarranted assumptions.

Social work educators stressed the need for students to recognize that individuals are not passive recipients of care; they make their own decisions:

They [the students] want to come and fix people and make things better. But, you know, people have choice. You know, some service users will say, ‘Actually I didn’t want that. You know, I have the right … to decline that service’. Sometimes students are horrified. ‘What do you mean that you said no?’ Well, because they’re an adult and they have choice. You can’t force things on people. (Participant 35, Programme Lead, BA)

Not everyone’s life is affected in the same way by the adversities they face and even when dealing with the most sensitive topics, some PwLE are willing to talk openly about issues that are extremely sensitive to broach with others.

So when you have people who have lived through it and survived it coming along and presenting the topic and giving you like, really frank details, I think that it’s good because it means that they [the students] don’t have any excuses to hide behind euphemisms and get shy about the issue. Because they’ve got the person in front of them who actually lived through it, who’s able to speak very openly about it that … it’s a good demonstration of how they should be seeing it and responding to it as well. (Participant 23, Graduate Teaching Assistant)

Finally, every educator to whom we spoke raised the connection between social work and human rights, social justice, service to humanity, and the importance of valuing and respecting people.

We’re encouraging students to … kind of get in touch more with the, the grass roots of what social work is. Yes, it’s an academic subject, but it’s also a practice-based profession ... [a] values led profession that is about human rights. It’s about social justice and within that there are lots of ethical complexities. And often the experts by experience who come to talk to us, they drill that home with the students best. (Participant 34, Assistant Professor)

## Discussion

In the UK, PwLE have helped to guide the design, delivery and review of social work curricula for two decades. This has created a wealth of practical insight into how, what and why involving PwLE contributes to social work education and professional development. Addressing a gap in the literature, we invited social work educators to reflect on the structure and content of the PwLE involvement in their courses and their perceptions of PwLE and student interactions. We interviewed educators at different career stages, from across Britain, teaching on a variety of undergraduate and post graduate courses and included some educators who had specific responsibilities for liaising with PwLE. Participation was, of course, voluntary and more likely to have been taken up by those with a particular interest in working with PwLE groups. We did not ask about the specific involvement models between the PwLE groups and institutions or programmes, nor did we apply one to the study. We did not interview students, who have been the focus of the evaluations mentioned in the introduction, nor the PwLE themselves, who are well-presented in the research literature.

Our findings suggest that educators agree that PwLE can convey vital components of the curriculum that are hard to access otherwise. The educators we talked to indicated numerous specific ways in which hearing direct experiences of social work and other services helpfully exposed the students to different perspectives, challenged stereotypes about people who access services, conveyed difficult and challenging information in a palatable and understandable format, demonstrated that the curriculum is connected to and grounded in the real world, and provided crucial preparation before practice. Our findings aligned with Rooney et al.’s (2019) study of staff perceptions of PwLE involvement at an English university, particularly in relation to PwLE creating a bridge between theory and practice, enabling students to critically reflect on their values and attitudes, and the perceived impact educators felt PwLE has on their pedagogic practice. Indeed, a thread throughout our data was the importance of complementary partnership work between educators and PwLE, for example through the incorporation of cognitive and affective learning, the bringing of up-to-date experiential knowledge by PwLE, and as has been reported by others (Driessens et al., 2016), by social work educators acting as a mediator between students and PwLE.

By working with PwLE, the staff themselves may reflect on their own understanding of social work practice and act as the exemplar to respectful collaboration and communication to students. Our study reinforces the messages from existing research on the necessity for educators to facilitate a support environment for PwLE to work within, engage with PwLE to actively consider and mitigate power differentials in the classroom, and to ensure participation by PwLE is not tokenistic (Fox, 2011; Molyneux & Irvine, 2004; Rooney et al., 2019). Nevertheless, educators not only have to reflect and act on their relationships with PwLE, but also ensure that the involvement is meaningfully embedded within learning objectives and aligned to the experience and study level of students (Robinson & Webber, 2013).

The creation of a clear, deliberate learning environment is therefore central to enabling effective interactions between PwLE and students (Tanner et al., 2017). Where the educators we interviewed described the involvement of PwLE in such terms, they closely linked this to a beneficial impact on students, particularly in terms of formative learning with regard to attitudes and assumptions. This correlates with the literature that explores students’ perspectives on the involvement of PwLE. Students across several studies have similarly described how teaching encounters with PwLE in the classroom have challenged their beliefs and prejudices, and thus facilitated the development of a meaningful understanding of social work values (Duffy, 2012; Hughes, 2017; Irvine et al., 2015). In our study, educators explained how engaging with PwLE meant students moved beyond ‘applying’ theory to individual ‘cases’. Instead, they were able to see how theory might be integrated into specific lived experiences.

A broader theme the educators in our study highlighted was around the importance of diversity of perspectives in social work education. Educators emphasized the role of multiple perspectives in preparing students to fully engage with complexity in practice. The inclusion of diverse characteristics, experiences and opinions has been evidenced as significant to learning and practice outcomes for students (Hughes, 2017). Conversely, limited representation can leave students with a distorted view of PwLE which in turn may reinforce negative attitudes (Anka & Taylor, 2016; Sapouna, 2021). Whilst broadly positive about the involvement of PwLE, the social work educators in our study did raise concerns about the limited diversity and representation of the PwLE individuals and groups that were linked to their courses; issues such as personal circumstances, dedication of time to participate, and difficulties with public speaking contributed to the challenge to find PwLE who could participate throughout the academic year and repeatedly speak about their (potentially traumatic) experience. A current challenge has been the Covid-19 pandemic, where educators reported the number of PwLE contributors decreasing due to lack of technology/devices to participate, the feeling of lack of inclusivity online than in a classroom, and personal circumstances that were exacerbated during the pandemic. Although not widely discussed in the interviews, the issues raised by educators do support research which suggests a broad range of PwLE experiences are not fully represented in social work; the difficulty of speaking to trauma highlights the complexity of involving the most stigmatized and marginalized groups in social work education (Burrows, 2012; Molyneux & Irvine, 2004).

In this regard, there are clear implications for social work research and pedagogical practice. Our study adds to the limited research which considers the view of social work educators on the involvement of PwLE in educating students. However, it also highlights questions which could be explored further, including educators’ perspectives on the range of PwLE who are involved in the social work programmes. In terms of characteristics, much of the current research is dominated by adult PwLE, with few studies reporting the involvement of children and young people (Lucas & Thomas, 2021). Broader questions of identity relating to ethnicity, culture, gender and sexuality are also underreported in the literature. Given the central role of social work educators in recruiting PwLE to contribute to their programmes, future research could focus on their views and theories on under (and over) representation, and the barriers to including a diversity of groups.

The educators in our study also understood the involvement of PwLE as equally vital as other forms of knowledge and highlighted the unique contribution that PwLE can make as part of a developed partnership. The implications for educators are that involving PwLE should be considered an integral element of programme design, rather an add-on requirement to learning which has already been designed around more traditional forms of knowledge and teaching methods. Embedding the contribution of PwLE on an equal footing with other ways of knowing, teaching and learning, acknowledges its critical role in an ‘ecological’ approach to understanding social work and those who access its services. It also acknowledges that social work is a fundamentally relationship-based profession that hinges on the professional’s ability to understand, empathize and connect with the experiences of those who use their services. It is clear from the literature that some barriers remain to embedding the involvement of PwLE in curriculum design and educational practice. Future research could fruitfully focus on possibilities for overcoming those barriers, and develop and evaluate innovative methods and designs for incorporating PwLE into social work education in embedded and sustainable ways.

## Conclusion

PwLE have been involved in commissioning, development and delivery of social work programmes in the UK for two decades. Social work educators see considerable value in including real-world accounts in teaching and learning. Social work educators believe that PwLE can help to connect the students’ learnings to the ‘real world’, using an applied approach that will help students reflect on their approaches to practice and their professional values. When social work educators see that pedagogic practice is clearly connected to the real world, students stand to gain a broad understanding of the ethics, values and empathy that is necessary to fully grasp their role as practitioner, inform their decisions, and understand the consequences for people who access social work services. Attention could now be more focused on diversifying representation of PWLE and sustainably embedding PwLE involvement in social work education programmes.

## Data Availability

All data, stimuli and analysis scripts belonging to this research are available through the APA repository in the Open Science Framework via the following link: https://doi.org/10.5281/zenodo.1212328

## Open Scholarship

This article has earned the Center for Open Science badge for Preregistered. The materials are openly accessible at https://doi.org/10.5281/zenodo.1212328
